# The role of the ATP-adenosine axis in ischemic stroke

**DOI:** 10.1007/s00281-023-00987-3

**Published:** 2023-03-14

**Authors:** Ines Sophie Schädlich, Riekje Winzer, Joschi Stabernack, Eva Tolosa, Tim Magnus, Björn Rissiek

**Affiliations:** 1 Department of Neurology, University Medical Centre Hamburg-Eppendorf, Martinistraße 52, 20246 Hamburg, Germany; 2 Institute of Immunology, University Medical Centre Hamburg-Eppendorf, Martinistraße 52, 20246 Hamburg, Germany

**Keywords:** Stroke, ATP, Adenosine, P2X7, Ectoenzymes

## Abstract

In ischemic stroke, the primary neuronal injury caused by the disruption of energy supply is further exacerbated by secondary sterile inflammation. The inflammatory cascade is largely initiated by the purine adenosine triphosphate (ATP) which is extensively released to the interstitial space during brain ischemia and functions as an extracellular danger signaling molecule. By engaging P2 receptors, extracellular ATP activates microglia leading to cytokine and chemokine production and subsequent immune cell recruitment from the periphery which further amplifies post-stroke inflammation. The ectonucleotidases CD39 and CD73 shape and balance the inflammatory environment by stepwise degrading extracellular ATP to adenosine which itself has neuroprotective and anti-inflammatory signaling properties. The neuroprotective effects of adenosine are mainly mediated through A_1_ receptors and inhibition of glutamatergic excitotoxicity, while the anti-inflammatory capacities of adenosine have been primarily attributed to A_2A_ receptor activation on infiltrating immune cells in the subacute phase after stroke. In this review, we summarize the current state of knowledge on the ATP-adenosine axis in ischemic stroke, discuss contradictory results, and point out potential pitfalls towards translating therapeutic approaches from rodent stroke models to human patients.

**Supplementary Information:**

The online version contains supplementary material available at 10.1007/s00281-023-00987-3.

## Introduction

Stroke ranks among the leading causes of death and disability worldwide. With 7.6 million new cases in 2019, ischemic stroke accounted for 62% of all strokes, and 3.3 million patients died from this widespread disease [1]. At present, treatment options of acute ischemic stroke are limited to intravenous (i.v.) thrombolysis and mechanical thrombectomy both aiming at reperfusion of the clotted vessel. Due to strict inclusion criteria, these therapies are only available for a subset of patients, and many patients present with persistent neurological deficits despite successful recanalization. Therefore, additional treatment strategies to combat ischemic brain injury are urgently needed. Research over the last three decades identified the substantial contribution of sterile inflammation to secondary neuronal injury following ischemic stroke. Consequently, targeting secondary infarct growth mediated by infiltrating immune cells emerged as a promising therapeutic strategy. The inflammatory cascade is initiated by the excessive release of danger-/damage-associated molecular patterns (DAMPs) which activate pattern recognition receptors on microglia and astrocytes with subsequent cytokine and chemokine production and immune cell attraction from the periphery [2]. Among these DAMPs, the purine adenosine triphosphate (ATP) plays a pivotal role. Geoffrey Burnstock pioneered the concept that ATP released by cells acts as an extracellular signaling molecule and thus exerts key functions beyond energy supply [3].

**Fig. 1:**
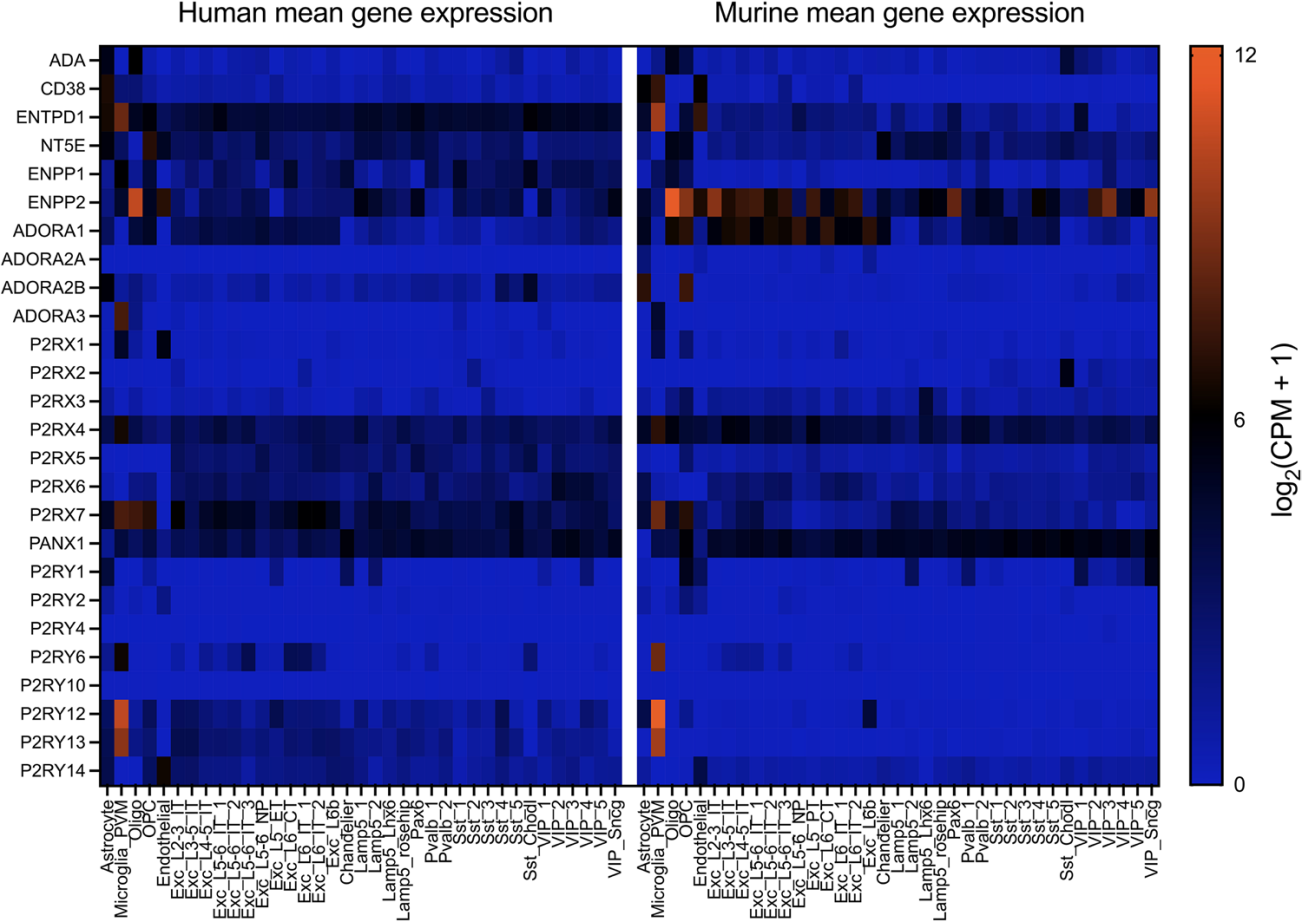
Gene expression data heatmap of 26 enzymes and receptors (y axis) involved in purinergic signaling in 37 conserved cell types (x axis) between humans (left) and mice (right). All enzymes and receptors are listed with gene names. For human single-nucleus RNA-sequencing data (SMART-SEQ) of the middle temporal gyrus (MTG), 15,928 nuclei from 8 human tissue donors were analyzed [10] (Allen Institute for Brain Science. Allen Brain Map, Cell Types Database, RNA-Seq Data. Available from: https://celltypes.brain-map.org/rnaseq/human/mtg). For the mouse cortex single-cell RNA-sequencing dataset (SMART-SEQ) 14,249 cells from the primary visual cortex (V1) and 9573 cells from the anterior lateral motor cortex (ALM) were profiled [11] (Allen Institute for Brain Science. Allen Brain Map, Cell Types Database, RNA-Seq Data. Available from: https://celltypes.brain-map.org/rnaseq/mouse/v1-alm). Expression levels were quantified as log_2_-transformed counts per million (CPM) of intronic plus exonic reads. Homologous cell types between the two species [10] can be divided into 5 non-neuronal cells (astrocytes, microglia and perivascular macrophages (PVM), oligodendrocytes (Oligo), oligodendrocyte precursor cells (OPC), and endothelial cells), 12 excitatory neuron (Exc) types named after the originating brain lamina (L) and their projection targets (intratelencephalic (IT), extratelencephalic-pyramidal tract (ET), near-projecting (NP), corticothalamic (CT)) and 20 inhibitory neuron cell types. Inhibitory neurons are further subdivided into lysosomal associated membrane protein family member 5 (Lamp5), somatostatin (Sst), parvalbumin (Pvalb), vasoactive intestinal polypeptide (Vip), and paired box 6 (Pax6) expressing cells and chandelier neurons

Under physiological conditions, cells within a tissue, including the brain, release ATP and nicotinamide adenine dinucleotide (NAD^+^) into the interstitial space in an activity-dependent and regulated manner. Release mechanisms include exocytosis, connexin or pannexin hemichannels, ATP-binding cassette (ABC) transporters, calcium homeostasis modulator (CALHM) channels, a macropore after P2X7 receptor overstimulation, maxi-anion channels (MACs), and volume-regulated ion channels (VRACs) as comprehensively reviewed by Giuliani et al. [4]. At sites of inflammation or ischemia, ATP is further liberated in an uncontrolled fashion via cell damage and death [4]. Indeed, an increase in extracellular ATP concentrations within the brain parenchyma during in vivo ischemia was measured by microdialysis [5, 6]. To improve the accuracy of extracellular ATP sensing and to circumvent methodological challenges of purine measurements within intact tissues, the group of Francesco Di Virgilio engineered a chimeric plasma membrane luciferase (pmeLUC) [7]. PmeLUC emits a measurable bioluminescent signal upon D-luciferin administration in the presence of extracellular ATP. Using this in vivo ATP biosensor, ATP release could be visualized as early as 30 min and up to 24 h after transient middle cerebral artery occlusion (tMCAO) [8]. After being released, ATP can either signal through its ionotropic P2X and metabotropic P2Y receptors or experience degradation to adenosine by ectoenzymes. The present review will give an overview about the different players of purinergic signaling, their expression in mice and humans (Fig. 1), and their contribution to tissue damage and regeneration in ischemic stroke. Details on the cited experimental stroke studies using mice with global genetic deficiency for the different players of purinergic signaling can be found in STab. 1.

### Signaling of ATP via P2X receptors in stroke

Extracellular ATP serves as ligand for all purinergic receptors of the ionotropic P2X family and for several P2Y receptors, while its metabolite ADP binds exclusively to P2Y receptors. The P2X family comprises seven members (P2X1-P2X7). Three P2X receptor subunits form an ion channel that upon ATP-mediated gating conducts the rapid influx of calcium and sodium ions and efflux of potassium ions. The expression of P2X receptors has been ascribed to various brain-resident cells as well as to cells of the immune system [9] with P2X1, P2X4, and P2X7 being associated with the outcome of cerebral ischemic events. In the central nervous system (CNS) of both mice and humans, microglia show the highest expression of these three receptors [10–13] (Fig. 1).

Of importance in the context of ischemic stroke, P2X1 is expressed on platelets. ATP (10 μM) increases platelet aggregation in vitro, which can be prevented by the P2X1 antagonist NF449 [14]. P2X1 is also expressed by neutrophil granulocytes of the mouse, where its activation promotes neutrophil chemotaxis [15]. Pharmacological blockade of P2X1 and genetic deletion of *P2rx1* in mice diminished the activation and recruitment of neutrophils to inflamed arteriolar endothelial cells, accompanied by impaired platelet aggregation and fibrin generation [16]. Therefore, targeting of P2X1 could be a promising new approach to ameliorate neutrophil driven post-stroke inflammation as well as stroke-associated thromboinflammation [17]. Apart from immune cells, P2X1 is also expressed on vascular smooth muscle cells (VSMCs) of rat middle cerebral arteries [18]. It has been shown that P2X1 and P2X4 form heterotrimers in VSMCs, which, in contrast to P2X1 homotrimers, contribute to ATP-mediated vasoconstriction. The impact of P2X1 signaling on VSMCs in the context of stroke still needs to be investigated.

ATP released during cerebral ischemia triggers P2X4 opening on brain innate immune cells such as microglia or infiltrating monocytes/macrophages and sustained P2X4 activation contributes to the ischemic injury [19, 20]. Among immune cells, P2X4 is expressed on T cells promoting early T cell activation in concert with P2X7 [21] as well as chemotaxis [22]. Indeed, P2rx4 deficiency in mice led to smaller infarcts after tMCAO [23]. Interestingly, when specifically deleting *P2rx4* in myeloid cells using LysM-Cre mice, only female mice showed a reduction in infarct size, suggesting a sex difference in the P2X4 response after stroke [23]. Another experimental stroke study by the same group verified the detrimental role of P2X4 in brain ischemia by using the P2X4 antagonist 5-BDBD: When applied 4 hours (h) after tMCAO, 5-BDBD reduced infarct size, number of infiltrating pro-inflammatory myeloid cells, and blood-brain-barrier (BBB) disruption [24]. 5-BDBD was also shown to attenuate brain injury in a mouse model of intracerebral hemorrhage, significantly reducing brain edema, BBB disruption, neuronal cell death, and neurological deficits [25]. Another study demonstrated that P2X4 is also expressed by pyramidal neurons of the rat hippocampal CA1 region [26]. In line with tMCAO data, intracerebroventricular (i.c.v.) 5-BDBD treatment immediately after cerebral ischemia (four-vessel occlusion model) and once 24 h later was also protective by blocking neuronal apoptosis, thereby promoting neuronal survival. Similar results were obtained with the P2X4 antagonist PSB-12062 [26]. Given the central role of P2X4 in inflammation, cell migration, and also pain, new P2X4 antagonists are continuously developed. The group of Kenneth A. Jacobson recently described the generation and evaluation of MRS4719 and MRS4596, two potent new P2X4 antagonists (IC50 0.503 and 1.38 μM for human P2X4, respectively) with neuroprotective effects in murine ischemic stroke (tMCAO): MRS4719 dose dependently reduced infarct size and brain atrophy 3 and 35 days post-stroke [27]. First P2X4-specific antagonistic monoclonal antibodies have been developed by AstraZeneca [28]. The affinity-optimized clone IgG#151-LO showed high selectivity for human P2X4 and induced potent and complete blockage of P2X4 currents. Inhibition of spinal P2X4 either by intrathecal delivery of anti-P2X4 antibodies or by systemic delivery of an anti-P2X4 bispecific antibody with enhanced blood–spinal cord barrier permeability achieved analgesia for 7 days in a mouse model of neuropathic pain [28]. Further, single domain antibodies (nanobodies) against P2X4 have been developed, but did not show P2X4-antagonizing properties [29]. In principle, evaluation of new biological-based P2X4-targeting drugs in the setting of brain ischemia would be desirable, as these usually exhibit good pharmacological properties in vivo and are well suited for cell-specific targeting of P2X4. Both factors might be important for the successful translation into treating stroke patients. Two studies highlight the need for a differentiated approach regarding timing and cell tropism: Sustained absence of P2X4 signaling, as occurring in P2X4ko mice, reduced infarct size after tMCAO, but myeloid cell-specific P2X4 deficiency led to more depressive behavior 30 days after stroke onset compared to recovering wild-type (WT) mice [23]. As another example of potentially detrimental effects of P2X4 blockade, brain endothelial cells acquire ischemic tolerance in a P2X4-dependent fashion as 5-BDBD treatment 15 min before ischemic preconditioning prevents this tolerization [30]. Therefore, biologics-based spatiotemporal targeting of P2X4 could be the key to future therapies focusing on P2X4 in stroke. However, crossing the BBB still remains a challenge for most antibody-based constructs, an obstacle that needs to be overcome by modern antibody engineering technology [31].

P2X7 is most likely the most extensively studied P2X receptor in brain ischemia. P2X7 activation is known as a potent trigger for the NLRP3 inflammasome formation in macrophages and microglia, leading to the release of pro-inflammatory interleukin-1β (IL-1β), a powerful driver of post-stroke inflammation [32–34]. Yet, deciphering the complex role of P2X7 during the interlinked processes of post-stroke inflammation, BBB breakdown and neuronal recovery remains challenging.

First studies by Nancy Rothwell’s group did not show a difference in stroke size when comparing *P2rx7*-deficient and WT mice [35], and i.c.v. treatment with P2X antagonists oxidized ATP and PPADS immediately before and again 30 minutes (min) after tMCAO did not affect lesion size after tMCAO either [36]. However, other studies demonstrated that intraperitoneal treatment with the broad P2 antagonist Reactive Blue 2 5 min after intraluminal permanent MCAO (pMCAO) in rats reduced brain damage and that MCAO led to an upregulation of P2X7 on microglia [37]. P2X7 upregulation had already been observed by earlier studies with hypertensive rats subjected to pMCAO by electrocoagulation [38]. Other studies suggested a protective role of P2X7 in stroke: I.c.v. injection of the P2X7-specific agonist BzATP into rats 1 h after tMCAO improved their motor functions when compared to mock-injected controls [39]. Studies using Brilliant Blue G (BBG) as P2X7 antagonist reported different outcomes regarding the role of P2X7 in rodent stroke models: The group of Carlos Matute reported that intraperitoneal BBG treatment 30 min after ischemia onset reduced the lesion size in rats subjected to tMCAO [40] and later reproduced their findings in mice [41]. The study by Caglayan and colleagues in 2017 again confirmed that i.c.v. injection of BBG reduced lesion size in mice when injected 30 min before tMCAO [42]. Similarly, intraperitoneal BBG administration also turned out to be protective in murine photothrombotic stroke when applied 1, 3, and 5 days after ischemia [43]. In contrast, a study, in which BBG was injected intraperitoneally (i.p.) daily for 3 days immediately after a short (15 min) tMCAO in mice, did not demonstrate a difference in lesion size between treatment and control group [44].

As for P2X7 targeting compounds, contrasting results have also been reported in studies using *P2rx7*-deficient mice. As mentioned above, first stroke experiments with P2X7ko mice did not reveal differences in stroke size [35]. Later in 2015, the group of Carlos Matute reported smaller stroke lesions (tMCAO) in P2X7ko mice when compared to WT [41]. In contrast, the group of Michael Schäfer reported reduced edema formation in P2X7ko mice 3 days after tMCAO, while stroke lesion size did not differ between P2X7ko and WT mice [45]. A recent study published by our group again suggested a detrimental role of P2X7 in stroke: Mice overexpressing P2X7 in cells that naturally express P2X7 [46] exhibited larger infarcts compared to WT controls. Further, i.c.v. injection of P2X7-blocking nanobodies [47] in mice directly before tMCAO significantly reduced lesion size compared to the control group [8]. This study represents the first approach to use highly specific biologicals to block P2X7 in the context of stroke. As mentioned above, the BBB constitutes a major obstacle for i.v. application of antibody-based drugs targeting brain-resident cells like microglia. Even for the small P2X7-specific nanobodies only high doses beyond 1 mg lead to detectable amounts of the nanobodies on brain microglia [48]. Interestingly, an alternative approach based on adeno-associated virus (AAV)-mediated muscle cell transduction followed by in vivo production of P2X7 antagonistic nanobodies led to the successful diffusion of the nanobodies into the brain where they could be detected on microglia for up to 120 days after transduction [48]. Since the role of P2X7 in long-term recovery after stroke still remains elusive, the AAV approach could be utilized to investigate this technically challenging question of high translational relevance.

When using antibody or nanobody-based therapeutics, one has to monitor for potential immune-related adverse events (irAE). Antibody-induced inflammation via complement activation or activation of natural killer (NK) cells and macrophages can be prevented by modifying the Fc-region of antibodies. Nanobody monomers lack Fc regions in principle but can be tailored as Fc-fusion proteins with desired or no Fc-related functions [49, 50]. To date, no adverse effects of P2X7 blockade by antibodies or nanobodies have been described. However, one has to keep in mind that P2X7 blockade can also potentially alter the susceptibility towards infections. Indeed, the P2X7 receptor antagonism can have both beneficial and deleterious effects depending on the type of pathogen, its virulence, and the severity of infection as reviewed in [51].

In general, though various studies suggest a role of P2X7 in stroke, many P2X7-related pathways and mechanisms that participate in post-stroke inflammation, thromboinflammation, BBB breakdown, neuroprotection and recovery remain to be revealed and connected. Contrasting results may be the consequence of (1) variations in the specificity of applied P2X7 antagonists, (2) different stroke models that do or do not allow reperfusion or that are based on photothrombosis, (3) differences related to administration routes or pre- versus post-treatment, or (4) the use of *P2rx7*-deficient mice of different origin. For the latter aspect, differences between the GlaxoSmithKline (GSK) and the Pfizer P2X7ko mice have already been demonstrated [52]: GSK P2X7ko mice lack P2X7 expression on innate immune cells but exhibit robust expression of P2X7 on T cells due to an escape splice variant. In contrast, the Pfizer P2X7ko mice lack P2X7 in both immune cell compartments. These differences might explain the opposite outcome of experimental autoimmune encephalomyelitis (EAE) studies: GSK P2X7ko mice showed suppressed EAE development compared to WT mice [53], whereas Pfizer P2X7ko mice exhibited an exacerbated disease course in EAE when compared to WT mice [54]. GSK P2X7ko mice have not been investigated in murine stroke models yet. Additionally, one has to keep in mind that the Pfizer P2X7ko mice are congenic and therefore might contain passenger mutations from 129 embryonic stem cells used for *P2rx7* gene targeting. Indeed, our group could identify a *P2rx7* passenger mutation in the commonly used P2X4ko mouse [55, 56]. Since *P2rx4* and *P2rx7* are direct neighboring genes, differentially expressed genes in congenic P2X4ko might also occur in congenic P2X7ko mice. If such passenger mutations also exist in the widely used Pfizer P2X7ko mouse [57] still needs to be investigated, in particular if they affect the outcome of stroke.

Of note, the ATP-gated P2X receptors expressed in the brain differ greatly in their sensitivity to ATP. As measured in transfected *X. laevis* oocytes or HEK293 cells by electrophysiology, the ATP concentration to induce the half-maximal response (*K*_1/2_) is about ten times lower for rat P2X1 (0.82 μM) than for rat P2X4 (11 μM). Rat P2X7, in turn, has a *K*_1/2_ of 130 μM and is accordingly about ten times less sensitive to ATP than P2X4 [58]. Thus, the activation of P2X receptors in the brain does not only depend on the expression pattern of the receptors, but also on the availability of extracellular ATP.

### Role of P2Y receptors in stroke

P2Y receptors are G-protein-coupled receptors activated by ATP, ADP, and other naturally occurring nucleoside phosphates. The most abundant P2Y receptors in the brain are P2Y12 and P2Y13, expressed at high levels in microglial cells (Fig. 1). The natural ligand for these two receptors is ADP, which upon engagement inhibits adenylate cyclase leading to a decrease in cyclic AMP (cAMP) and subsequent activation of microglia. P2Y12 receptor inhibitors, such as clopidogrel and ticagrelor, are commonly used for the secondary prevention of atherosclerotic ischemic stroke because of their effective reduction of P2Y12-mediated platelet aggregation. However, the beneficial effects of these inhibitors are not limited to this anti-thrombotic effect, since they are also neuroprotective in murine tMCAO (oral administration from 24 h before to 8 h after occlusion) [59], anti-atherosclerotic [60], and can reduce microglial activation and chemotaxis after pMCAO in rats (oral treatment 10 min, 22 and 36 h after occlusion) [61]. Interestingly, physiological concentrations of ADP facilitate the contact of microglia processes with neuronal cell bodies. After neuronal injury, higher concentrations of ADP triggered microglial-mediated neuroprotection in a P2Y12 receptor-dependent manner, sparing viable neurons from cell death and maintaining functional connectivity [62]. These data pose a warning to the treatment with P2Y12 inhibitors for stroke prevention and prompt the analysis of neuronal function in MCAO models under P2Y12 inhibitor treatment.

ADP can also activate P2Y1 receptors, expressed in astrocytes, and is a partial agonist for the P2Y6 receptor, expressed by microglial cells. While pre- and post-stroke blockade of P2Y1 by MRS2500 i.c.v. proved beneficial for stroke outcome in transient and permanent MCAO models [63, 64], the role of P2Y6 in stroke is still controversial: Pharmacological inhibition of P2Y6-mediated phagocytosis with MRS2578 on 3 consecutive days after tMCAO aggravated infarct size, brain atrophy, and neurological deficits [65]. However, in a model of brain ischemia induced by endothelin-1 injection, knockdown of the P2Y6 receptor had a beneficial effect by delaying phagocytosis of stressed neurons in the peri-infarct region [66]. P2Y2 and P2Y11 receptors are not expressed on brain-resident cells but are present on immune cells that can be attracted to the brain after ischemia-reperfusion injury. Indeed, ATP signaling through P2Y2 and P2Y11 promotes neutrophil and monocyte migration [67–69]. Therefore, a transient increase of systemic ATP after stroke may contribute to brain immune cell infiltration. Of note, ATP has antagonistic effects on P2Y12 [70], which may constitute an intrinsic regulatory mechanism to prevent extended inflammation.

### Ectoenzymes convert extracellular ATP via ADP and AMP to adenosine

Extracellular ATP has a half-life of less than 5 min. Ectonucleotidases sequentially degrade extracellular ATP to dampen the pro-inflammatory effects of ATP signaling and to generate adenosine as a metabolite with signaling properties itself [4]. Once produced, adenosine signals through four different G-protein-coupled P1 receptors and has neuroprotective [71] and anti-inflammatory [72] properties. Modulation of ischemic brain injury by adenosine signaling will be elucidated in the next section.

**Fig. 2:**
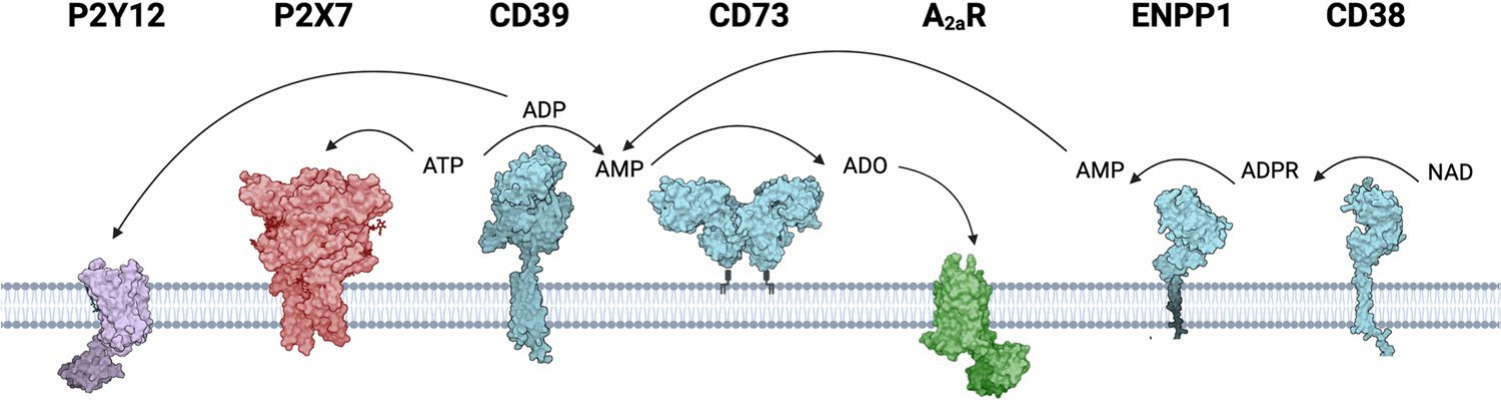
Nucleotide-metabolizing enzymes. Extracellular ATP serves as ligand for ionotropic P2X receptors, e.g., P2X7. On the canonical pathway, ATP is degraded to ADP and AMP by the ectonucleotidase CD39. ADP is a natural ligand for P2Y12. CD73 finally dephosphorylates AMP to generate adenosine (ADO). On the alternative non-canonical pathway, AMP is generated from NAD^+^: CD38 hydrolyzes NAD^+^ to generate ADPR, which is further converted to AMP by ENPP1. Extracellular adenosine serves as ligand for adenosine receptors, e.g., A_2A_R

On the canonical pathway of adenosine production (Fig. 2), ecto-nucleoside triphosphate diphosphohydrolase 1 (NTPDase1/CD39) hydrolyzes ATP to adenosine diphosphate (ADP) and then to adenosine monophosphate (AMP). AMP is metabolized to adenosine by ecto-5’-nucleotidase (NT5E/CD73) which is the dominant adenosine-generating enzyme. CD73 activity can be inhibited by high concentrations of ATP and ADP [73]. The alternative non-canonical pathway to generate AMP for subsequent adenosine formation independently of CD39 involves NAD glycohydrolase/CD38 and ectonucleotide pyrophosphatase/phosphodiesterase 1 (ENPP1/CD203a): CD38 hydrolyzes extracellular NAD^+^ to ADP ribose (ADPR) which is further converted to AMP by ENPP1 [74]. On top of that, tissue non-specific alkaline phosphatase (TNAP) mainly expressed at the vasculature contributes to adenosine formation in the rodent and human brain by catalyzing all steps in the degradation of nucleoside 5’-tri-, -di-, and -monophosphates to adenosine [75, 76].

In mouse and human brain, CD39 expression is highest on microglia followed by endothelial cells [10–13, 76] (Fig. 1). In the immune cell compartment it is expressed by neutrophils, monocytes, dendritic cells (DCs), B cells, regulatory T cells (Tregs), and NK cells (Immunological Genome Project). In the mouse brain, CD73 expression is highest on oligodendrocytes whereas both astrocytes and oligodendrocytes show high expression in humans [10–13] (Fig. 1). It has to be noted that, in contrast to human brain endothelial cells [77], CD73 could not be detected on mouse brain endothelial cells in vivo but shows high expression in the olfactory bulb and in the caudoputamen [76, 78, 79]. In the immune cell compartment CD73 is expressed by T and B cells, neutrophils, and macrophages (Immunological Genome Project). CD38 expression is highest on astrocytes followed by endothelial cells in both mice and humans, whereas ENPP1 is mainly expressed by microglia in both species and by oligodendrocytes in mice [10–13] (Fig. 1). The enzyme adenosine deaminase (ADA) completes the purine-inactivating machinery and catalyzes the deamination of adenosine to inosine. The highest tissue ADA enzyme activity in humans was found in lymphoid tissues, particularly the thymus, the brain, and the gastrointestinal tract [80]. In the rodent brain, immunohistochemistry revealed an extensive plexus of ADA-containing neurons in the hypothalamus [81]. Genetic deficiency of ADA2 results in an autoimmune syndrome characterized by systemic vasculitis and inflammation. Ischemic strokes occur early in childhood and are often a consequence of systemic vasculitis affecting the CNS, but stroke can also represent the initial clinical symptom without any signs of cerebral vasculitis [82, 83].

Taken together, ectonucleotidases shape the inflammatory microenvironment by tightly controlling and balancing the extracellular concentration of pro-inflammatory ATP and anti-inflammatory adenosine.

In the late 1990s, CD39 was identified as a potent thromboregulator by rapidly degrading ADP released from activated platelets at sites of vascular injury, thus inhibiting ADP-induced platelet activation, recruitment, and aggregation [84–86]. Consistent with the notion that platelet and fibrin accumulation occurs distal to the occluded vessel leading to microvascular thrombosis [87], CD39 knockout mice exhibited increased cerebral infarct volumes and reduced post-ischemic reperfusion after tMCAO [88], whereas treatment of WT mice with recombinant soluble human CD39 up to 3 h after induction of stroke reduced infarct sizes at 24 h and promoted an increase in post-ischemic blood flow [88]. Global transgenic overexpression of CD39 in mice was likewise protective in photothrombotic stroke [89]. Selective CD39 overexpression in myeloid lineage cells also conferred a reduction in infarct volume indicating a contribution of the ATP-degrading machinery on infiltrating immune cells to the protective phenotype [89]. However, these experiments were only conducted in a small number of animals.

Regarding the role of CD73 in ischemic stroke, contradictory results can be found in the literature. While mice deficient for CD73 exhibited larger infarcts and increased immune cell infiltration into the ischemic hemisphere than wild-type mice after photothrombotic stroke [79], global CD73 deficiency did not affect infarct volume, immune cell infiltration, and glia cell activation profiles following ischemia-reperfusion injury induced by tMCAO in our laboratory [90]. It seems most plausible that these conflicting findings can be attributed to the different experimental stroke models used: In the photothrombotic stroke model, focal activation of the photosensitive dye induces severe endothelial cell injury, local thrombus formation, and rapid blood-brain barrier breakdown leading to pronounced vascular (extracellular) edema. The irreversibly injured infarct core is not surrounded by functionally impaired tissue predisposed for infarction but capable of surviving, the so-called penumbra, and the vessel is permanently occluded not allowing reperfusion. On the contrary, ischemic stroke induced by tMCAO is characterized by a leading cytotoxic (intracellular) edema, a penumbra with delayed neuronal cell death due to secondary mechanisms such as inflammation and reperfusion [91].

When interpreting and extrapolating results on CD73 from experimental stroke studies conducted in mice, one has to keep in mind that CD73 is absent on murine brain endothelial cells but highly expressed on peripheral endothelial cells and human brain endothelial cells [76, 77, 92] (Fig. 1). These expression differences account for the finding that CD73 knockout mice exhibited increased vascular leakage in peripheral organs where CD73-derived adenosine signaling is implicated in endothelial barrier function via A_2A_ and A_2B_ adenosine receptors (AR), whereas vascular permeability in the brain was not affected [93].

Beyond brain-resident cells, infiltrating immune cells play a central role in secondary neuronal injury, and their CD73 expression can also influence stroke outcome. Murine CD4^+^CD25^+^Foxp3^+^ Tregs co-express CD39 and CD73, and adenosine generated by their coordinated activity mediates both their immunosuppressive effects on activated T effector cells and their own expansion and functionality through A_2A_R signaling [94, 95]. In vivo expansion of Tregs with interleukin-2/interleukin-2 antibody (IL-2/IL-2Ab) complex reduced infarct volume and infiltration of T cells and macrophages into the ischemic hemisphere after tMCAO. Mechanistically, CD73 expression on Tregs played a crucial role in IL-2/IL-2Ab-afforded protection as Tregs prepared from CD73 knockout mice with or without IL-2/IL-2Ab treatment failed to protect WT-recipient mice in an adoptive transfer experiment [96]. Analogous to Treg functions, Th17 responses are also shaped by CD73 activity [4]. On γδ T cells, low CD73 expression correlated with enhanced Th17-response-promoting activity [97]. Given that IL-17A-producing γδ T cells promote early detrimental neutrophil infiltration after stroke [98], modulating their CD73 expression may attenuate post-stroke inflammation.

The ectoenzyme CD38 catalyzes, inter alia, the conversion of NAD^+^ to ADPR on the alternative pathway of adenosine formation and controls NAD^+^ bioavailability and activity of NAD-dependent enzymes in the brain as reviewed by Guerreiro et al. [99]. Besides, chemotaxis of myeloid cells in response to different chemoattractants depends on CD38 and its products ADPR and cyclic ADPR [100, 101]. Several studies demonstrated protective effects of CD38 deficiency in animal models of neurodegeneration and neuroinflammation [99]. Regarding ischemic stroke, CD38 knockout mice showed smaller infarcts after tMCAO [102] and reduced hippocampal neuronal cell death after transient forebrain ischemia due to occlusion of both common carotid arteries and concomitant reduction of mean arterial blood pressure [103]. CD38 knockout mice also showed a drastic reduction of macrophage recruitment to the ischemic hemisphere which probably contributed to the protective phenotype [102]. Beneficial effects of CD38 deletion in brain pathologies were additionally attributed to increased NAD^+^ levels itself [99, 104] as it has strong neuroprotective and anti-inflammatory properties [105]. NAD^+^ depletion, on the other hand, leads to mitochondrial dysfunction and oxidative stress and is a common finding in neurodegenerative disorders [99, 106]. CD38 expression and enzymatic activity were recently shown to increase with age in the rat brain [107] complementing previous findings on an age-dependent decrease in brain NAD^+^ levels in healthy humans [108]. Spontaneously hypertensive stroke-prone rats, the most frequently used animal model for cerebral small vessel disease leading to lacunar infarction and cognitive decline, also exhibited higher CD38 expression and activity in the brain than age-matched normotensive controls [107]. Taken together, there is growing indirect evidence that CD38 might link aging with neuroinflammation and neurodegeneration and thus remains an interesting target in stroke.

### Role of adenosine receptors in cerebral ischemia

The concentration of extracellular adenosine increases substantially during in vivo ischemia. By microdialysis technique, Melani et al. measured a basal concentration of 130 nmol/l in the rat striatum which increased 10-fold within the first hour after intraluminal pMCAO and then decreased again but remained elevated until the end of the observation period 4 h after stroke induction [6, 109]. The half-life of extracellular adenosine is very short (4–10 s), as it is swiftly degraded by adenosine deaminase to its metabolite inosine. Therefore, to maintain an increased level of adenosine implies a continuous production. The increase of extracellular adenosine during hypoxia mainly relies on two mechanisms: Within the first 20 min of ischemia, adenosine derives mostly from hydrolysis of extracellular ATP involving CD73 leading to a higher extracellular than intracellular adenosine concentration [6]. Thereafter, extracellular adenosine originates mainly from parenchymal cells where it accumulates in a pO_2_-dependent manner [110]. Consequently, the gradient between extracellular and intracellular adenosine concentrations is reversed, and adenosine is released from the cells to the extracellular space [6, 111]. Under hypoxic conditions, mitochondria consume ATP, and accumulating AMP cannot be reconverted to ATP due to the lack of oxygen and glucose. Hence, ATP and ADP levels decrease below the threshold to inhibit cytosolic 5’-nucleotidase [112], and adenosine is excessively formed from AMP. Intracellular adenosine concentrations further rise due to the hypoxia-induced inhibition of adenosine kinase which removes adenosine by phosphorylation into AMP and represents another key enzyme for the regulation of ambient adenosine levels [113–115]. Given that all these cellular and enzymatic processes determine adenosine concentration, caution should be taken in extrapolating tissue adenosine levels from measurements of CD73 mRNA or protein expression only [111].

**Fig. 3:**
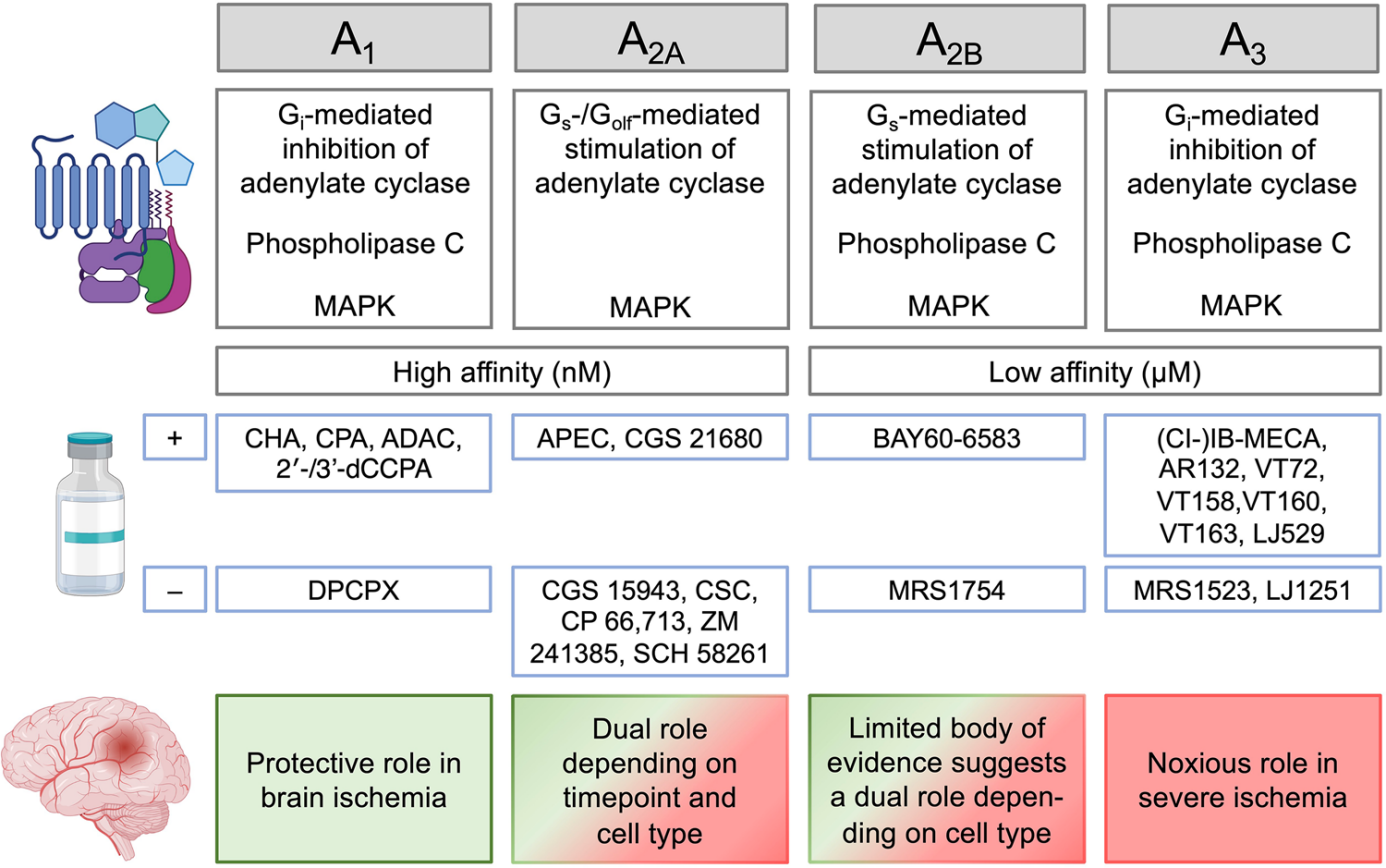
Overview on the signaling pathways and affinity of the four known subtypes of adenosine receptors (ARs), stimulatory (+) and inhibitory (–) compounds used in the cited studies on OGD or in vivo ischemic stroke and their role in brain ischemia. For details, see Supplementary Table 2. Images from BioRender.com

Adenosine release during ischemia is considered an endogenous neuroprotective response as adenosine infusion through a microdialysis probe into the rat striatum reduced neurological deficits after tMCAO [116]. Pre- and post-ischemic (2 h) i.v. delivery of adenosine-containing nanoparticles allowing prolonged circulation of the nucleoside also resulted in a significant improvement of infarct volume and neurologic deficit score after transient and permanent intraluminal MCAO [117]. Administration of an adenosine kinase inhibitor likewise attenuated ischemic damage in rats after tMCAO [118], whereas transgenic overexpression of adenosine kinase in the murine brain leads to increased infarct volumes after tMCAO [119]. Given that targeted delivery of adenosine to the CNS is hampered by a very short half-life in plasma, low blood-brain barrier permeability [120], and major systemic side effects such as bradycardia via cardiac A_1_R [121], therapeutic approaches focused on the modulation of adenosine receptor signaling. The A_1_, A_2A_, A_2B_, and A_3_ receptors are the four known AR subtypes, which all belong to the superfamily of G-protein-coupled receptors and differ regarding their affinity for adenosine and tissue distribution. Apart from inhibition or stimulation of adenylate cyclase, signaling occurs through phospholipase C (PLC), calcium, and mitogen-activated protein kinases (MAPK) [122] (Fig. 3). At the concentration range during physiologic conditions, adenosine can stimulate the high affinity adenosine receptors A_1_ and A_2A_, but during ischemia, adenosine reaches concentrations in the low micromolar range sufficient to also activate low affinity A_2B_ and A_3_R [123]. A_1_R shows an overall higher expression in mice than in humans with a quite even distribution across neurons in cortex, hippocampus, and cerebellum, oligodendrocytes, and astrocytes. At the cellular level, A_2A_R expression is highest on endothelial cells followed by astrocytes in both species, and at the spatial level, A_2A_R density is highest at post-synaptic neurons in the striatum. In the striatum, A_2A_Rs mainly signal through activation of stimulatory G_olf_ [124]. While astrocytes followed by murine OPCs show the highest expression of A_2B_R in the CNS, A_3_R expression at the cellular level is highest on microglia in both mice and humans and at the spatial level most dense on hippocampal neurons [10–13, 125] (Fig. 1). The main approach in the development of AR agonists has been the chemical modification of adenosine itself, whereas AR antagonists are mainly derived from xanthines such as caffeine and theophylline [122]. A review by Pedata et al. comprehensively summarized our current knowledge on the role of all four adenosine receptors in brain ischemia both in vitro and in vivo [126]. In the present review, we will therefore only give a condensed overview with focus on data from in vivo stroke studies. STab. 2 provides detailed information for all the cited studies using pharmacological modulation of AR signaling with focus on common quantitative outcome measures of translational relevance, e.g., infarct volume and neurological outcome.

A_1_R signaling in response to ischemia exerts protective effects by inhibiting excitatory synaptic transmission [127–129], in particular glutamate, which is crucial for the functional recovery of hippocampal circuits upon reoxygenation [130–132]. Acute systemic administration of different A_1_R agonists (CHA, CPA, ADAC) was protective in a model of global cerebral ischemia in gerbils [133–135], whereas acute treatment with the antagonist DPCPX exacerbated ischemic brain injury [134]. Of note, the effects of chronic administration, i.e., pre-treatment for several days before the induction of stroke, are opposite [134]. It has been suggested that ischemic protection by chronic administration of adenosine antagonists may be a consequence of A_1_R upregulation and that detrimental effects of chronic A_1_R agonism are due to the phenomenon of A_1_R desensitization [136] which has been described for rat hippocampal slices upon hypoxia [137]. A_1_R signaling also plays a central role in mediating the beneficial effects of ischemic preconditioning in the brain [138, 139], a phenomenon in which the previous exposure to a short period of ischemia confers resistance to a following prolonged ischemic insult. To overcome the limitations of full A_1_R agonists for clinical application, mainly receptor desensitization and cardiovascular side effects such as bradycardia and hypotension, the neuroprotective potential of partial agonists (2′-dCCPA, 3′-dCCPA) was recently investigated and proven in oxygen-glucose deprivation (OGD) as the most frequently used in vitro model of cerebral ischemia [140].

Protective effects of A_2A_R antagonism (CGS 15943) on ischemic injury were first demonstrated in 1994 by Gao and Phillis after global forebrain ischemia in gerbils [141]. In the following years, many studies using different models of rodent experimental stroke (global forebrain ischemia, four-vessel occlusion, intraluminal pMCAO, pMCAO by electrocoagulation) confirmed the beneficial role of various A_2A_R antagonists—namely, CSC, CP 66,713, ZM 241385, SCH 58261—when being administered before ischemia [142, 143] or acutely after stroke [144–146]. The latter three studies on SCH 58261 evaluated infarct volume at 24 h after stroke. Later, Melani et al. demonstrated that ischemic protection by SCH 58261 was limited to outcome parameters on day 1, whereas prolonged treatment for 7 days did not confer sustained reduction in infarct volume [147]. In line with the findings on drug-mediated antagonism, mice genetically deficient for A_2A_R exhibited smaller infarcts 2 h, 24 h, and 48 h after tMCAO [148, 149] and 24 h after embolic MCAO [150]. Interestingly, RNA-sequencing analysis of the ipsilesional and contralesional primary motor cortices (iM1 and cM1) 15 days after tMCAO revealed that downregulation of *Adora2a* encoding A_2A_R in cM1 correlated with improved recovery [151]. Detrimental effects of A_2A_R signaling are largely attributed to an increase of extracellular glutamate concentrations leading to excitotoxicity, thus counteracting the depression of excitatory transmission mediated by A_1_R [126]. Several mechanisms contribute to glutamatergic excitatory transmission upon A_2A_R stimulation: First, it increases neuronal glutamate release during ischemia in vitro [152, 153], and genetic A_2A_R deficiency was shown to reduce striatal glutamate outflow measured by microdialysis during tMCAO and reperfusion [148]. Second, A_2A_R activation on astrocytes inhibits glutamate uptake [154–157]. Third, A_1_ and A_2A_R can form heteromers on striatal glutamatergic nerve terminals in which A_2A_R activation reduces the affinity of the A_1_R for agonists shifting the balance towards A_2A_R effects in an adenosine-rich milieu such as ischemia [158].

Beyond the control of glutamatergic excitotoxicity, A_2A_R antagonists exert their protective effects through inhibition of phospho-p38 MAPK [145] and by reducing JNK MAPK activation in oligodendrocytes [159]. Moreover, A_2A_R activation was shown to drive microglia process retraction into an amoeboid shape upon activation which represents a hallmark of neuroinflammation and modulates their phagocytotic capacities [160]. A_2A_R signaling on brain endothelial cells contributes to ischemic brain injury as endothelium-specific A_2A_R-deficient mice also showed reduced infarct volumes 24 h after stroke [150].

When given systemically and chronically 13 days before global brain ischemia or until day 7 after tMCAO, A_2A_R agonists (APEC, CGS 21680) also conferred protection [161, 162]. These findings seem paradoxically but rely on different mechanisms than protection by antagonists. While central effects like regulation of cerebral blood flow [161] and control of neurotrophic factors [163] might account in part for this phenomenon, protection is mainly attributed to immunomodulatory effects of A_2A_R signaling. The substantial contribution of sterile inflammation to secondary neuronal injury is nowadays widely accepted and summarized elsewhere [2, 164, 165]. A_2A_R is abundantly expressed on innate and adaptive immune cells, and its activation serves as a strong anti-inflammatory signal [166, 167]. A_2A_Rs even increase in density and affinity on lymphocytes from ischemic stroke patients indicating the translational relevance of this pathway [168]. It has to be noted that the A_2A_R agonist CGS 21680 only conferred protection when being chronically administered, whereas delivery limited to the first 24 h after stroke did not reduce infarct volume [169] as it probably missed the peak of immune cell infiltration at day 3 after tMCAO [170].

Taken together, research over the last decades demonstrated a dual role of A_2A_R signaling in brain ischemia depending on the timepoint and the leading mechanism of injury: A_2A_R antagonists reduce ischemic injury early after stroke by reducing excitotoxicity, whereas A_2A_R agonists confer protection when being administered for several days post-ischemia by attenuating secondary sterile inflammation. From a translational perspective, therapeutic strategies with antagonists or agonists at the A_2A_R should therefore be carefully evaluated in terms of timing.

Compared to A_2A_R signaling, far less literature is available on the role of A_2B_R signaling in ischemic stroke. Though ubiquitously expressed [171], the low affinity of A_2B_R for its endogenous ligand adenosine and the lack of potent and selective agonists and antagonists has hampered investigations into its specific function in health and disease for a long time. Moreover, the role of A_2B_R is often masked by co-expression of A_2A_R, which especially holds true for monocytes and macrophages, where A_2A_R remains the predominant adenosine receptor. Due to recent innovations regarding pharmacological tools and molecular approaches, A_2B_Rs are increasingly recognized as orchestrators of immunity and inflammation as reviewed by Haskó and colleagues [172]. In the immune cell compartment, A_2B_R activation increases pro-inflammatory cytokine production by mast cells, whereas it shapes the function and cytokine profile of antigen-presenting cells such as DCs and macrophages towards an anti-inflammatory and tolerance-inducing phenotype [172]. A_2B_R knockout mice displayed a mild but significant increase in pro-inflammatory cytokines under basal conditions [171].

On endothelial cells, A_2B_R signaling plays a central role in controlling barrier function. The protective effect is even potentiated under hypoxic conditions because of adenosine accumulation and A_2B_R upregulation [173, 174]. Consequently, A_2B_R knockout mice exhibited increased vascular leakage measured by Evans’s blue extravasation in multiple organs including the brain during hypoxia, whereas A_1_R, A_2A_R, and A_3_R deficiency did not accentuate hypoxia-induced vascular leakage compared to wild-type littermate controls [175]. A_2B_R knockout mice expressed higher levels of adhesion molecules at the peripheral vasculature resulting in higher numbers of leukocytes adhering at the endothelium under basal conditions [171] and increased infiltration of polymorphonuclear leukocytes in various organs including the brain after hypoxia [175]. Bone marrow transplantation experiments demonstrated that A_2B_R signaling on hematopoietic cells mainly accounted for A_2B_R-mediated attenuation of hypoxia-associated neutrophil transmigration, while vascular A_2B_R conferred protection to fluid leakage [171, 175].

A recent in vivo study in rats demonstrated that systemic and chronic treatment with the A_2B_R agonist BAY60-6583 after tMCAO diminished immune cell infiltration at day 2 and reduced infarct volume at day 7 after stroke [176]. These protective effects of A_2B_R signaling are contrasted by a study indicating a rather detrimental role of central A_2B_R signaling: In transient global brain ischemia induced by four-vessel occlusion in rats, the selective A_2B_R antagonist MRS1754 reduced the phosphorylation of p38 MAPK and subsequent activation of neutral sphingomyelinase 2 which otherwise leads to pro-inflammatory ceramide accumulation in hippocampal astrocytes [177]. Taken together, central A_2B_R signaling on astrocytes seems to exert a pro-inflammatory role, while A_2B_R activation on immune cells and endothelial cells dampens inflammation and vascular leakage.

Several in vivo stroke studies demonstrated a protective effect of A_3_R agonists: Pre-treatment with the A_3_R agonist CI-IB-MECA either once i.c.v. 15 min or twice i.v. 165 min and 15 min before transient MCA ligation reduced infarct volume at day 2 in rats and mice [178]. In the same study, A_3_R knockout mice exhibited larger infarcts after tMCAO than WT controls [178]. With higher relevance from a translational perspective, post-treatment with agonists also proved effective: Administration of the A_3_R agonist LJ529 twice within the first seven hours after tMCAO reduced infarct volume at day 1 in rats. This effect was only reversed by application of the A_3_R antagonist MRS1523 but not by co-administration of an A_2A_R antagonist (SCH 58261) indicating that ischemic protection was indeed mediated by A_3_R [179]. Recently, delivery of the A_1_/A_3_R agonist AST-004 via bolus and continuous infusion starting 2 h after tMCAO limited the lesion growth rate measured by repeated magnetic resonance imaging (MRI) scans and overall stroke volume at day 5 in macaques as an example of non-human primates [180]. In vitro data from various OGD experiments seemed to be contradictory regarding the effects of A_3_R signaling on synaptic transmission and excitotoxicity during ischemia with both agonists and antagonists exerting ischemic protection under different experimental conditions [126]. In 2007, Pugliese and colleagues deciphered the divergent contribution of A_3_R signaling depending on the severity of OGD in rat hippocampal slices: While A_3_R agonists had an acute A_1_R-like inhibitory effect on synaptic transmission during a short period of OGD (2–5 min), both antagonists (LJ1251, MRS1523) and prolonged exposure to agonists (CI-IB-MECA, VT72, VT158, VT160, VT163, AR132) prevented or delayed anoxic depolarization and were protective during severe OGD (7 min) [181]. It is assumed that severe ischemia switches A_3_R-mediated effects from beneficial to detrimental via sustained activation of PLC and protein kinase C through G_q_ proteins and subsequent calcium mobilization. The yet protective effects of chronic A_3_R agonist treatment are a consequence of receptor desensitization in an per se adenosine-rich microenvironment [181], which has been shown for both rat and human A_3_R [182, 183].

This noxious role of A_3_R signaling in conditions of severe ischemia explains early findings by von Lubitz and colleagues, who showed that acute treatment with the A_3_R agonist IB-MECA right before global forebrain ischemia increased mortality until day 7 after ischemia in gerbils, whereas chronic pre-treatment over 10 days, probably leading to A_3_R desensitization, enhanced neuronal survival in the hippocampus [184].

Beyond receptor desensitization, the protective effects of A_3_R agonists are also mediated by immune cells as demonstrated by Choi et al. [179]. The A_3_R agonist LJ529 when given twice at 2 h and 7 h after tMCAO reduced the migration of activated microglia and infiltration of monocytes into ischemic cortical and striatal lesions. In a microglial cell culture, LJ529 inhibited chemotaxis of microglia and monocytes, and co-treatment with A_3_ and A_2A_R antagonists indicated that this effect was selectively mediated by A_3_R [179]. A_3_R was found to be overexpressed in inflammatory tissue and on peripheral blood mononuclear cells from patients with different autoimmune inflammatory diseases such as rheumatoid arthritis, psoriasis, and Crohn’s disease [185, 186]. Various A_3_R agonists exerted anti-inflammatory effects across different experimental models of inflammation [187] which is mediated by de-regulation of the NF-kB signaling pathway inhibiting cytokine production and enhancing apoptosis of inflammatory cells [186]. The highly selective A_3_R agonist CF101 even proved effective and safe in a phase II clinical trial in rheumatoid arthritis [188]. If the evidence for a protective role of A_3_R agonists in ischemic stroke mounts, CF101 might indeed represent an interesting therapeutic perspective.

Taken together, A_3_R signaling takes on a noxious role in conditions of severe ischemia such as long OGD and in vivo stroke, and the yet protective effects of chronic A_3_R agonist treatment are mainly attributed to receptor desensitization.

### Differences between murine and human purinergic signaling pathways

Mice and humans differ in their expression and regulation of P2 receptors, particularly with respect to P2X7. P2X7 is expressed at lower levels in humans (at least on circulating T cells) than in mice, as shown, for example, for Tregs, which have a very high P2X7 expression in mice, but low expression in humans [189, 190]. Compared to rodent P2X7, human P2X7 is around ten times less sensitive to ATP [58] and requires ATP concentrations in the high micromolar to low millimolar range for activation [191]. Additionally, only murine P2X7 is activated by low NAD^+^ concentrations through ARTC2.2-mediated ADP ribosylation, as there is no human orthologue of ARTC2.2 [192, 193]. P2Y11, an ATP-gated G-protein-coupled receptor, is expressed in humans, but not in mice. P2Y11 interacts with P2X7 and inhibits P2X7-mediated pore formation and cell death [194]. Overall, the hurdle to activate P2X7 appears to be significantly higher in humans than in mice.

Major differences between mice and humans also exist in the ectonucleotidases degrading ATP to adenosine. As mentioned earlier, CD73 is highly expressed on human, but not on murine brain endothelial cells [76, 77, 92]. This could strongly influence brain adenosine concentrations at the site of ischemic stroke. Unlike hypoxia-induced upregulation of CD73 on human microvascular endothelial cells in vitro [173], our group did not detect an upregulation of CD73 on murine cerebral microvessels after ischemic stroke [90]. The expression of CD73 on infiltrating immune cells also differs between the two species. Murine Tregs express both CD39 and CD73 on the cell surface, allowing the generation of adenosine from ATP by the concerted action of both ectonucleotidases [94]. In contrast, human Tregs exhibit very low CD73 expression [195]. Similarly, almost all conventional T cells in mice express CD73, whereas human CD4 and CD8 cells (except for naïve CD8 T cells and a subset of memory cells) have low CD73 expression [195, 196]. A reason for the differential expression of CD73 in mice and humans may be that adenosine receptor signaling has different effects in the two species. In contrast to mice, where adenosine promotes Treg expansion, adenosine signaling in humans inhibits the activation of Tregs [95, 197–199]. The absence of CD73 on Tregs could prevent adenosine-mediated auto-inhibition. Adenosine is degraded by ADA, which in humans, but not in mice, is “anchored” to the plasma membrane by the ectonucleotidase CD26. ADA in close proximity to the cell surface could reduce the autocrine effect of adenosine in humans.

Due to these differences in purinergic signaling between mice and humans, results from mouse models need to be carefully evaluated and are unlikely to be directly applicable to humans.

### Concluding remark

A large number of preclinical studies clearly demonstrated the involvement of the ATP-adenosine axis in stroke pathophysiology reaching from excitotoxicity over post-stroke inflammation to recovery. From today’s perspective, the translation of drugs targeting key players of purinergic signaling from experimental stroke research into clinical routine is mainly hampered by (i) the BBB that constitutes a major obstacle for the i.v. application of antibody-based therapeutic constructs, (ii) a potentially dual role of certain targets depending on the timepoint and leading mechanism of injury (e.g., A_2A_R), (iii) major cardiovascular side effects (e.g., A_1_R agonists), (iv) gene expression differences between mice and humans (e.g., CD73), and (v) futility of pre-treatment strategies in human stroke. Especially when aiming at reducing post-stroke inflammation, e.g., through P2X7 blockade, the rapid onset of P2X7 signaling after stroke will probably leave a narrow time window for treatment. To our opinion, the engineering of P2X7-blocking nanobodies with improved ability to cross the BBB after i.v. injection could be a huge step towards translation. Further, the application of nanobodies via the intranasal route could be a promising therapeutic approach. A high dose of P2X7-blocking nanobodies could be given as nasal spray at the first signs of stroke. The successful distribution of nanobodies via the nasal route targeting, e.g., transthyretin in the CNS has already been demonstrated in mice [200]. Apart from targeting P2X7, the use of partial AR agonists offers the greatest therapeutic potential. Nevertheless, any therapeutic approach must be carefully evaluated in terms of timing, efficiency irrespective of age and gender, specificity, and effects on long-term outcome.

## Supplementary information

Supplementary materials 1: Supplementary Table 1 Knockout mice for purinergic signaling investigated in brain ischemia. Supplementary Table 2 Pharmacological modification of AR signaling in brain ischemia. (DOCX 80 kb)

## Abbreviations

AAV: Adeno-associated virus
ADA: Adenosine deaminase
ADAC: Adenosine amine congener
ADP: Adenosine diphosphate
ADPR: ADP ribose
AMP: Adenosine monophosphate
APEC: 2-[(2-Aminoethylamino)-carbonylethylphenylethylamino]-5′-N-ethylcarboxoamidoadenosine
AR: Adenosine receptor
AR132: N^6^-Methyl-2-phenylethynyladenosine
ATP: Adenosine triphosphate
BAY60-6583: 2-[[6-Amino-3,5-dicyano-4-[4-(cyclopropylmethoxy) phenyl]-2-pyridinyl] thio]-acetamide
BBB: Blood-brain-barrier
BBG: Brilliant Blue G
5-BDBD: 5-(3-Bromophenyl)-1,3-dihydro-2H-benzofuro[3,2-e]-1,4-diazepin-2-one
BzATP: 2'-3'-O-(4-benzoylbenzoyl) adenosine 5'-triphosphate
cAMP: Cyclic AMP
CGS 15943: 9-Chloro-2-(2-furanyl)-[1,2,4]triazolo[1,5-c]quinazolin-5-amine
CGS 21680: 2-p-(2-Carboxyethyl)-phenethylamino-5′-N-ethylcarboxamidoadenosine
CHA: Cyclohexyladenosine
CI-IB-MECA: Chloro-N6-(3-iodo-benzyl)-adenosine-5′-N-methyluronamide
cM1: Contralesional primary motor cortex
CNS: Central nervous system
CPA: N6-cyclopentyladenosine
CP 66,713: 4-Amino-l-phenyl[1,2,4]-triazolo[4,3-a] quinoxaline
CSC: 8-(3-Chlorostyryl) caffeine
DAMPs: Danger-/damage-associated molecular patterns
DCs: Dendritic cells
2′-dCCPA: 2-Chloro-2′-deoxy-N6-cyclopentyladenosine
3′-dCCPA: 2-Chloro-3′-deoxy-N6-cyclopentyladenosine
DPCPX: 1,3-Dipropyl-8-cyclopentylxanthine
EAE: Experimental autoimmune encephalomyelitis
ENPP1/CD203a: Ectonucleotide pyrophosphatase/phosphodiesterase 1
GSK: GlaxoSmithKline
h: Hours
i.c.v.: Intracerebroventricular
IL-2/IL-2Ab: Interleukin-2/interleukin-2 antibody
iM1: Ispilesional primary motor cortex
i.p.: Intraperitoneal
irAE: Immune-related adverse event
i.v.: Intravenous
LJ529: 2-Chloro-N^6^-(3-iodobenzyl)-5′-N-methylcarbamoyl-4′-thioadenosine
LJ1251: (2R,3R,4S)-2-(2-chloro-6-(3-iodobenzylamino)-9H-purin-9-yl)tetrahydrothiophene-3,4-diol
MAPK: Mitogen-activated protein kinase
*K*_1/2_: Half-maximal response
min: Minutes
MRI: Magnetic resonance imaging
MRS1523: 3-Propyl-6-ethyl-5-[(ethylthio)carbonyl]-2-phenyl-4-propyl-3-pyridine carboxylate
MRS1754: 8-[4-[((4-Cyanophenyl)carbamoylmethyl)oxy]phenyl]-1,3-di(n-propyl) xanthine hydrate
MRS2500: (1R*,2S*)-4-[2-iodo-6-(methylamino)-9H-purin-9-yl]-2-(phosphono-oxy)bicycle[3.1.0] hexane-1-methanol dihydrogen phosphate ester
MRS2578: 1,4-Di[3-(3-isothiocyanatophenyl)thioureido]butane
MRS4719: 5-(2-(5-Thioxo-4,5-dihydro-1,2,4-oxadiazol-3-yl)pyridin-4-yl)-1,5-dihydro-2H-naphtho[1,2-b][1,4]diazepine-2,4(3H)-dione triethylamine salt
NAD^+^: Nicotinamide adenine dinucleotide
NF449: 4,4',4",4"'-(Carbonylbis (imino-5,1,3-benzenetriylbis (carbonylimino)))tetrakis-benzene-1,3-disulfonic acid
NK cells: Natural killer cells
NTPDase1/CD39: Ecto-nucleoside triphosphate diphosphohydrolase 1
NT5E/CD73: Ecto-5’-nucleotidase
OGD: Oxygen-glucose deprivation
PLC: Phospholipase C
pMCAO: Permanent middle cerebral artery occlusion
pmeLUC: Plasma membrane luciferase
PPADS: Pyridoxalphosphate-6-azophenyl-2′,4′-disulphonic acid
SCH 58261: 7-(2-Phenylethyl)-5-amino-2-(2-furyl)-pyrazolo-[4,3-e]-1,2,4-triazolo[1,5-c]pyrimidine
tMCAO: Transient middle cerebral artery occlusion
TNAP: Tissue non-specific alkaline phosphatase
Tregs: Regulatory T cells
WT: Wild type
VSMCs: Vascular smooth muscle cells
VT72: N6-Methoxy-2-phenylethynyl
VT158: N6-Methoxy-2-phenylethynyl
VT160: N6-Methoxy-2-(2-pyridinyl)-ethynyl
VT163: N6-Methoxy-2-p-acetylphenylethynyl
ZM 241385: 4-(-2-[7-Amino-2-{2-furyl}{1,2,4}triazolo{2,3-a}{1,3,5}triazin-5-yl-amino]ethyl)phenol
