# Supplementary material for: The role of the ATP-adenosine axis in ischemic stroke
Source: Semin Immunopathol. 2023 Mar 14;45(3):347–65. doi: 10.1007/s00281-023-00987-3 (PMC10279578; doi:10.1007/s00281-023-00987-3)
Supplement: Supplementary file 1 — Supplementary Table 1 Knockout mice for purinergic signaling investigated in brain ischemia. Supplementary Table 2 Pharmacological modification of AR signaling in brain ischemia. (DOCX 80 kb) [file 281_2023_987_MOESM1_ESM.docx]

**Supplementary Table 1** Knockout mice for purinergic signaling investigated in brain ischemia.

| **Knockout mouse line** | **Age & gender** | **Stroke model** | **Outcome parameters** | **Effect** | **REF** |
| --- | --- | --- | --- | --- | --- |
| *P2rx4*^–/–^  (MGI:6117070) | 20-25 g,  male & female | 60 min tMCAO | Infarct volume at day 3 (cresyl violet) | Reduction (both sexes) | [23] |
|  |  |  | Brain atrophy at day 30 (cresyl violet) | No effect (both sexes) |  |
|  |  |  | Neurological outcome at day 2 (motor score) | Improvement (both sexes) |  |
|  |  |  | Neurological outcome at day 28 (motor score) | No effect (both sexes) |  |
|  |  |  | Microglia activation at day 3 (Iba 1, morphology) | Reduction |  |
| *P2rx7*^–/–^  (MGI: n/a) | n/a | 30 min tMCAO | Infarct volume at 24 h (cresyl violet) | No effect | [35] |
|  |  | 60 min tMCAO |  | No effect |  |
|  |  | pMCAO (intraluminal) |  | No effect |  |
| *P2rx7*^–/–^  (MGI: n/a) | 25-32 g, male | 60 min tMCAO | Infarct volume at day 3 (TTC) | Reduction | [41] |
|  |  |  | Neurological outcome at day 1 and 3 (Bederson score) | Improvement |  |
|  |  |  | Neuronal survival in the infarct core at day 3 (Fluoro-Jade C) | Improvement |  |
| *P2rx7*^–/–^  (MGI:2386080) | 25-35 g, male | 60 min tMCAO | Infarct volume at day 1 and 3 (MRI: T2, ADC, DWI) | No effect | [45] |
|  |  |  | Brain swelling at day 1 and 3 (MRI: midline shift) | Increase at day 1 |  |
|  |  |  | Neurological outcome at 6 h, day 1, 2, 3 (motor score) | No effect |  |
|  |  |  | Microglia activation at day 3 (Iba1) | Reduction |  |
| *Entpd1^–/–^*  (MGI:2673318) | n/a | 45 min tMCAO | Infarct volume at 24 h (TTC) | Increase | [88] |
|  |  |  | Neurological outcome at day 1 (motor score) | No effect |  |
| *Nt5e^–/–^*  (MGI:3522017) | 10 weeks, male | Photothrombotic stroke | Infarct volume at day 2 (MRI: T2) | Increase | [79] |
|  |  |  | Neurological outcome at day 2 (motor score) | Deterioration |  |
|  |  |  | Immune cell infiltration into the ischemic hemisphere at day 2 (flow cytometry) | Increase (microglia, macrophages, neutrophils, T cells) |  |
| *Nt5e^–/–^*  (MGI:6257868) | 11-16 weeks, male | 50 min tMCAO | Infarct volume at day 3 (TTC) | No effect | [90] |
|  |  |  | Immune cell infiltration into the ischemic hemisphere at day 3 (flow cytometry) | No effect (microglia, macrophages, neutrophils, T cells) |  |
|  |  |  | Glia cell gene expression profile at day 3 (NanoString) | No effect |  |

| *Cd38^–/–^*  (MGI:1861803) | 12-15 weeks, male | 60 min tMCAO | Infarct volume at day 3 (TTC) | Reduction | [102] |
| --- | --- | --- | --- | --- | --- |
|  |  |  | Neurological outcome at day 3 (motor score) | Improvement |  |
|  |  |  | Immune cell infiltration into the ischemic hemisphere at day 3 (flow cytometry) | Reduction (T cells, macrophages) |  |
| *Cd38^–/–^*  (MGI: n/a) | 3 months, male | 10 min bilateral carotid occlusion + reduction of mean arterial blood pressure | Neuronal survival in hippocampal CA1 region at day 6 (cresyl violet) | Improvement | [103] |
| *Adora2a^–/–^*  (MGI:2155985) | 8-10 weeks, n/a | 2 h tMCAO | Infarct volume at 2 h, 24 h and 48 h (cresyl violet) | Reduction | [148] |
|  |  |  | Neurological outcome at 2 h, 24 h and 48 h  (motor score) | Improvement |  |
|  |  |  | Mean glutamate outflow during 2 h MCAO  (striatal microdialysis) | Reduction |  |
| *Adora2a^–/–^*  (MGI:2155985) | 18-25 g,  male & female | 2 h tMCAO | Infarct volume at 24 h (TTC, H&E) | Reduction | [149] |
|  |  |  | Neurological outcome at 24 h (motor score) | Improvement |  |
| *Adora2a^–/–^*  (MGI:2155985) | 14-16 weeks, male | Embolic MCAO | Infarct volume at 24 h (TTC) | Reduction | [150] |
|  |  |  | Neurological outcome at 24 h (motor score) | Improvement |  |
| *Adora3^–/–^*  (MGI:2155986) | n/a, male | 90 min tMCAO | Infarct volume at day 2 (TTC) | Increase | [178] |

**Supplementary Table 2** Pharmacological modification of AR signaling in brain ischemia.

| **Pharmacological agent** | **Agonist / antagonist** | **Dosage** | **Species** | **Stroke model** | **Treatment regime** | **Outcome parameters** | **Effect** | **REF** |
| --- | --- | --- | --- | --- | --- | --- | --- | --- |
| **A_1_R** | | | | | | | | |
| CHA | Agonist | 2 mg/kg i.p. | Gerbil, female,  70-80 g | 30 min bilateral carotid occlusion (forebrain ischemia) | Single dose 5 min post-reperfusion | Survival at day 14 | Improvement | [133] |
|  |  |  |  |  |  | Neurological outcome at day 14 (composite score) |  |  |
| CPA | Agonist | 1 mg/kg i.p. | Gerbil, female,  60-70 g | 10 min bilateral carotid occlusion | Single dose 15 min prior to occlusion (acute) | Survival at day 7 day | Improvement | [134] |
|  |  |  |  |  |  | Neuronal survival in hippocampal CA1 region at day 7 (Nissl staining) |  |  |
|  |  |  |  |  | Daily injection for 15 days followed by 1 drug-free day before occlusion (chronic) | Survival at day 7 | Deterioration |  |
|  |  |  |  |  |  | Neuronal survival in hippocampal CA1 region at day 7 (Nissl staining) |  |  |
| ADAC | Agonist | 100 µg/kg i.p. | Gerbil, female,  70 g | 5 min bilateral carotid occlusion | Single dose 15 min, 30 min, 1, 2, 3, 6, 12 or 18 h post-reperfusion | Survival at day 14 | No death in any group | [135] |
|  |  |  |  |  |  | Neuronal survival in hippocampal CA1 region at day 7 (Nissl staining) | Improvement for treatment at 6 and 12 h |  |
|  |  |  |  | 10 min bilateral carotid occlusion | Single dose 15 min, 30 min, 1, 2, 3, 6, 12 or 18 h post-reperfusion | Survival at day 14 | Improvement for treatment within 15 min to 2 h |  |
|  |  |  |  |  |  | Neuronal survival in hippocampal CA1 region at day 7 (Nissl staining) | Improvement for all treatment timepoints (best effect for 1 h) |  |
|  |  |  |  |  |  | Spatial memory and learning capacity at day 13 (Morris water maze) | Improvement for treatment at 6 h (not at 12 h) |  |

| 2’-dCCPA  3’-dCCPA | Partial agonist | 0.1, 0.5, 1, 10 µM | Human neuro-blastoma cells (SH-SY5Y) | 4 h OGD *in vitro*,  24 h recovery | 1 h prior to OGD until the end of the recovery period | Cell viability at 24 h (Trypan blue staining) | Improvement at concentration of 0.5 and 1 µM (both) and 10 µM (2’-dCCPA only) | [140] |
| --- | --- | --- | --- | --- | --- | --- | --- | --- |
|  |  | 2 µM | Hippocampal slices  (C57Bl/6 mouse, male,  8-16 weeks) | 7 min OGD  *in vitro* | 10 min before OGD | CA1 hippocampal neurotransmission (fEPSP) | Recovery of neurotransmission |  |
| DPCPX | Antagonist | 1 mg/kg i.p. | Gerbil, female,  60-70 g | 10 min bilateral carotid occlusion | Single dose 15 min prior to occlusion (acute) | Survival at day 7 | Deterioration | [134] |
|  |  |  |  |  |  | Neuronal survival in hippocampal CA1 region at day 7 (Nissl staining) |  |  |
|  |  |  |  |  | Daily injection for 15 days followed by 1 drug-free day before occlusion (chronic) | Survival at 7 days | Improvement to the same extent as with acute CPA treatment |  |
|  |  |  |  |  |  | Neuronal survival in hippocampal CA1 region at day 7 (Nissl staining) |  |  |
|  |  | 0.1 mg/kg i.p. | Gerbil,  male,  50-60 g | 5 min bilateral carotid occlusion | Single dose 20 min prior to ischemia | Neuronal survival in hippocampal CA1 region at day 5 (cresyl violet) | No effect | [142] |
|  |  | 1 mg/kg i.p. |  |  |  |  | Deterioration |  |
|  |  | 0.1 mg/kg i.p. | Wistar rat, male,  8-9 weeks | 180 min tMCAO | Single dose 95 min prior to occlusion | Infarct volume at day 7 (TTC) | No effect | [138] |
|  |  |  |  |  |  | Neurological outcome at day 2, 4 and 7 (Bederson score) | Deterioration at day 7 |  |
|  |  |  |  | 30 min tMCAO (preconditioning), 60 min reperfusion,  180 min tMCAO | Single dose 5 min prior to preconditioning | Infarct volume at day 7 (TTC) | Removal of the protective effects of pre-conditioning |  |
|  |  |  |  |  |  | Neurological outcome at day 2, 4 and 7 (Bederson score) |  |  |
|  |  | 0.1 mg/kg i.p. | Wistar rat, male,  8-9 weeks | 120 min tMCAO | Single dose 30 min prior to occlusion | Infarct volume at day 7 (H&E) | No effect | [139] |
|  |  |  |  | 30 min tMCAO (preconditioning), 3 d reperfusion,  120 min tMCAO | Single dose 30 min prior to pre-conditioning | Infarct volume at day 7 (H&E) | Removal of the protective effects of pre-conditioning |  |
|  |  |  |  |  | Single dose 30 min prior to 2^nd^ occlusion |  | Sustained protection by preconditioning |  |
| **A_2A_R** | | | | | | | | |
| APEC | Agonist | 0.1 mg/kg i.p. | Gerbil, female,  70 g | 10 min bilateral carotid occlusion | Single dose 15 min prior to ischemia (acute) | Cortical blood flow until 90 min after reperfusion (laser Doppler) | Improvement | [161] |
|  |  |  |  |  |  | Survival at day 14 | Improvement |  |
|  |  |  |  |  |  | Neuronal survival in hippocampal CA1 region at day 15 (Nissl staining) | No effect |  |
|  |  |  |  |  | Daily injections for 13 days, last injection 15 min prior to ischemia on day 14 (chronic) | Cortical blood flow until 90 min after reperfusion (laser Doppler) | Improvement |  |
|  |  |  |  |  |  | Survival at day 14 | Improvement |  |
|  |  |  |  |  |  | Neuronal survival in hippocampal CA1 region at day 15 (Nissl staining) | Improvement |  |
| CGS 21680 | Agonist | 0.01 or 0.1 mg/kg i.p. | Wistar rat, male,  270-290 g | 1 h tMCAO | Twice daily starting at 4 h post-reperfusion for 7 days | Neurological outcome at day 1, 5 and 7 (composite score) | Improvement  (both dosages) | [162] |
|  |  |  |  |  |  | Cortical infarct volume at day 7 (cresyl violet) | Reduction  (both dosages) |  |
|  |  |  |  |  |  | Striatal infarct volume at day 7 (cresyl violet) | No effect |  |
|  |  |  |  |  |  | Granulocyte infiltration at day 2 (HIS-48 staining) | Reduction in the cortex (only 0.01 mg/kg investigated) |  |
|  |  | 0.1 mg/kg i.p. | Wistar rat, n/a, n/a | pMCAO | Twice at 4 and 20 h post-reperfusion | Infarct volume at 24 h | No effect | [169] |
|  |  |  |  | tMCAO (n/a min) |  | Infarct volume at day 7 | No effect |  |
| CGS 15943 | Antagonist | 0.1 mg/kg i.p. | Gerbil, male,  50-55 g | 5 min bilateral carotid occlusion | Single dose 15 min prior to ischemia | Neuronal survival in hippocampal CA1 region at day 5 (cresyl violet) | Improvement | [141] |

| CSC | Antagonist | 1 mg/kg i.p. | Gerbil, female,  70 g | 10 min bilateral carotid occlusion | Single dose 15 min prior to ischemia (acute) | Cortical blood flow until 90 min after reperfusion (laser Doppler) | No effect | [161] |
| --- | --- | --- | --- | --- | --- | --- | --- | --- |
|  |  |  |  |  |  | Survival at day 14 | No effect |  |
|  |  |  |  |  |  | Neuronal survival in hippocampal CA1 region at day 15 (Nissl staining) | Improvement |  |
|  |  |  |  |  | Daily injections for 13 days, last injection 15 min prior to ischemia on day 14 (chronic) | Cortical blood flow until 90 min after reperfusion (laser Doppler) | Improvement |  |
|  |  |  |  |  |  | Survival at day 14 | No effect |  |
|  |  |  |  |  |  | Neuronal survival in hippocampal CA1 region at day 15 (Nissl staining) | No effect |  |
|  |  | 0.1 mg/kg i.p. | Gerbil, male,  50-60 g | 5 min bilateral carotid occlusion | Single dose 20 min prior to ischemia | Neuronal survival in hippocampal CA1 region at day 5 (cresyl violet) | Improvement | [142] |
|  |  | 1 mg/kg i.p. |  |  |  |  | No effect |  |
| CP 66,713 | Antagonist | 0.1 mg/kg i.p. | Gerbil, male,  50-60 g | 5 min bilateral carotid occlusion | Single dose 20 min prior to ischemia | Neuronal survival in hippocampal CA1 region at day 5 (cresyl violet) | Improvement | [142] |
|  |  | 1 mg/kg i.p. |  |  |  |  | Deterioration |  |
| ZM 241385 | Antagonist | 1 mg/kg i.p. | Wistar rat, male,  300-400 g | 10 min four-vessel occlusion under normo-glycemic and hyperglycemic conditions | Single dose 30 min prior to ischemia | Spatial memory and learning capacity at days 6, 7 and 8 (Morris water maze) | Improvement (hyperglycemic only) | [143] |
|  |  |  |  |  |  | Neuronal survival in hippocampal CA1, CA3 and hilum regions at day 8 (cresyl violet) | Improvement (hyperglycemic only) |  |
| SCH 58261 | Antagonist | 0.01 mg/kg i.p. | Wistar rat, male,  270-290 g | pMCAO (intraluminal) | Single dose 5 min post-occlusion | Outflow of excitatory amino acids from 0 to 240 min after MCAO (striatal microdialysis) | Reduction (glutamate, aspartate, adenosine, GABA) | [144] |
|  |  |  |  |  |  | Cortical infarct volume at 24 h (cresyl violet) | Reduction |  |
|  |  |  |  |  |  | Striatal infarct volume at 24 h (cresyl violet) | No effect |  |
|  |  |  |  |  |  | Sensorimotor deficit at 24 h (composite score) | No effect |  |
| SCH 58261  (*continued*) | Antagonist | 0.01 mg/kg i.p. | Wistar rat, male,  270-290 g | pMCAO (intraluminal) | Repeated injections  5 min, 6 h and 15 h post-occlusion | Infarct volume at 24 h (cresyl violet) | Reduction (cortical and striatal) | [145] |
|  |  |  |  |  |  | Sensorimotor deficit at 24 h (composite score) | Improvement |  |
|  |  |  |  |  |  | p38 MAPK phosphorylation at 24 h (western blot, IHC) | Reduction |  |
|  |  | 0.01 mg/kg i.v. or i.p. | Sprague-Dawley rat and SHR, male,  250-300 g | pMCAO (electro-coagulation) | Single dose 10 min post-occlusion | Cortical infarct volume at 24 h (TTC) | Reduction (i.v. and i.p.) in normotensive and hypertensive animals (SHR) | [146] |
|  |  | 0.01 mg/kg i.p. | Wistar rat, male,  270-290 g | 1h tMCAO | Repeated injections twice daily for 7 d starting 5 min post-occlusion | Neurological outcome at 24 h, 5 and 7 days (composite score) | Improvement only at 24 h | [147] |
|  |  |  |  |  |  | Immune cell infiltration at day 2 (HIS-48 staining) | No effect |  |
|  |  |  |  |  |  | Infarct volume at day 7 (cresyl violet) | No effect (neither cortical nor striatal) |  |
|  |  | 70 µg/kg i.p. | Sprague-Dawley rat, male,  260-270 g | 90 min tMCAO | Twice 2 and 7 h post-occlusion | Infarct volume at 24 h (TTC) | No effect | [179] |
| **A_2B_R** | | | | | | | | |
| BAY60-6583 | Agonist | 0.1 mg/kg i.p. | Wistar rat, male,  270-290 g | 1h tMCAO | Repeated injections twice daily for 7 d starting 4 h post-occlusion | Neurological outcome at 24 h, 5 and 7 days (composite score) | Improvement | [176] |
|  |  |  |  |  |  | Infarct volume at day 7 (cresyl violet) | Reduction (cortical and striatal) |  |
|  |  |  |  |  |  | Neuronal survival in the striatum at day 7 (NeuN) | Improvement |  |
|  |  |  |  |  |  | Granulocyte infiltration at day 2 (HIS-48 staining) | Reduction (cortical and striatal) |  |
| MRS1754 | Antagonist | i.c.v., no dosage information | Sprague-Dawley rat, male,  220-250 g | 10 min four-vessel occlusion | Single dose 30 min prior to occlusion | p38 MAPK phosphory-lation (western blot) and hippocampal ceramide levels (IHC) at 30 min | Reduction | [177] |
| **A_3_R** | | | | | | | | |
| (CI-)IB-MECA | Agonist | 4 µg/20µl i.c.v. | Sprague-Dawley rat, male,  age n/a | 60 min tMCAO + bilateral common carotid ligation | Sigle dose 15 min prior to occlusion | Infarct volume at day 2 (TTC) | Reduction | [178] |
|  |  | 0.2 mg/kg i.v. |  |  | Two doses 165 and 15 min prior to occlusion | Infarct volume at day 2 (TTC) | Reduction |  |
|  |  |  |  |  |  | Cortical cell survival (TUNEL) at day 2 | Improvement |  |
|  |  | 1 µg/5 µl i.c.v. | C57Bl/6 mouse, male,  age n/a | 90 min tMCAO | Sigle dose 15 min prior to occlusion | Infarct volume at day 2 (TTC) | Reduction |  |
|  |  | 100 µg/kg i.p. | Gerbil, female,  60-70 g | 10 min bilateral carotid occlusion | Single dose 15 min prior to occlusion (acute) | Survival at day 7 | Deterioration | [184] |
|  |  |  |  |  |  | Neuronal survival in hippocampal CA1 region at day 7 (Nissl staining) | n/a (only n=1 survived) |  |
|  |  |  |  |  | Repeated daily injections for 10 days followed by 1 drug-free day prior to occlusion (chronic) | Survival at day 7 | Improvement |  |
|  |  |  |  |  |  | Neuronal survival in hippocampal CA1 region at day 7 (Nissl staining) | Improvement |  |
|  |  |  |  | 20 min bilateral carotid occlusion | Single dose 15 min prior to occlusion (acute) | Survival at day 7 | Deterioration (all died before day 7) |  |
|  |  |  |  |  | Repeated daily injections for 10 days followed by 1 drug-free day prior to occlusion (chronic) | Survival at day 7 | Improvement |  |
|  |  |  |  |  |  | Neuronal survival in hippocampal CA1 region at day 7 (Nissl staining) | Improvement |  |
|  |  | 10 nM | Hippocampal slices  (Wister rat, male,  150-200 g) | 2 min OGD  *in vitro* | 5 min before, during, and 2 min after OGD | CA1 hippocampal neurotransmission (fEPSP) | Lower depression | [181] |
|  |  |  |  |  | 2 min during OGD |  | No effect |  |
|  |  |  |  | 5 min OGD  *in vitro* | 5 min before, during, and 2 min after OGD | CA1 hippocampal neurotransmission (fEPSP) | Faster recovery |  |
|  |  |  |  |  | 2 min at the end of the OGD period |  | Delayed recovery |  |
|  |  |  |  | 7 min OGD  *in vitro* | 5 min before, during, and 2 min after OGD | CA1 hippocampal neurotransmission (fEPSP) | Faster recovery, pre-vention/delay of anoxic depolarization (AD) |  |
| AR132 | Agonist | 10 nM | Hippocampal slices  (Wister rat, male,  150-200 g) | 7 min OGD  *in vitro* | 5 min before, during, and 2 min after OGD | CA1 hippocampal neurotransmission (fEPSP) | Faster recovery, prevention/delay of AD | [181] |
| VT72 |  | 10 nM |  |  |  |  |  |  |
| VT158 |  | 5 nM |  |  |  |  |  |  |
| VT160 |  | 5 nM |  |  |  |  |  |  |
| VT163 |  | 5 nM |  |  |  |  |  |  |
| AR132 | Agonist | 10 nM |  | 2 min OGD  *in vitro* | 2 min during OGD | CA1 hippocampal neurotransmission (fEPSP) | No effect | [181] |
| VT158 |  | 5 nM |  |  |  |  |  |  |
| VT160 |  | 5 nM |  | 5 min OGD  *in vitro* | 2 min at the end of the OGD period |  | Delayed recovery |  |
| LJ529 | Agonist | 2 mg/kg i.p. | Sprague-Dawley rat, male,  260-270 g | 90 min tMCAO | Twice 2 and 7 h post-occlusion | Infarct volume at 24 h (TTC) | Reduction | [179] |
|  |  |  |  |  |  | Microglia/monocyte infiltration at 24 h (ED1-staining) | Reduction (cortical and striatal) |  |
| MRS1523 | Antagonist | 100 nM | Hippocampal slices  (Wister rat, male,  150-200 g) | 2 min OGD  *in vitro* | 10 min before, during, and 5 min after OGD | CA1 hippocampal neurotransmission (fEPSP) | Lower depression | [181] |
|  |  |  |  | 5 min OGD  *in vitro* | 10 min before, during, and 5 min after OGD | CA1 hippocampal neurotransmission (fEPSP) | Faster recovery |  |
|  |  |  |  | 7 min OGD  *in vitro* | 10 min before, during, and 5 min after OGD | CA1 hippocampal neurotransmission (fEPSP) | Faster recovery, prevention of AD |  |
|  |  | 1 mg/kg i.p. | Sprague-Dawley rat, male,  260-270 g | 90 min tMCAO | Twice 2 and 7 h post-occlusion | Infarct volume at 24 h (TTC) | No effect | [179] |
| LJ1251 | Antagonist | 10 nM | Hippocampal slices  (Wister rat, male,  150-200 g) | 7 min OGD  *in vitro* | 10 min before, during, and 5 min after OGD | CA1 hippocampal neurotransmission (fEPSP) | Faster recovery, prevention of AD | [181] |

| **A_1_R & A_3_R** | | | | | | | | |
| --- | --- | --- | --- | --- | --- | --- | --- | --- |
| AST-004 | Agonist | 1.7 mg/kg + 0.9 mg/kg/h i.v. (mid dose)  5.2 mg/kg + 2.8 mg/kg/h i.v. (high dose) | Macaque, male,  47-83 months | 4 h tMCAO | Bolus 2 h post-occlusion (i.e. 2 h pre-reperfusion) followed by 22 h of continuous infusion | Ischemic lesion growth rate from 1.8 to 6 h post-occlusion (MRI: DWI) | Reduction | [180] |
|  |  |  |  |  |  | Infarct volume at 24 h (MRI: DWI) | No effect |  |
|  |  |  |  |  |  | Infarct volume at 5 d (MRI: DWI) | Reduction |  |
